# Inclusion of Milk Thistle Seed and *Achyranthes japonica* Extract Alone or in Combination in Diet of Weaning Pigs Results in Similar Growth Outcomes

**DOI:** 10.3390/life15071114

**Published:** 2025-07-16

**Authors:** Shanmugam Suresh Kumar, Se Yeon Jang, In Ho Kim

**Affiliations:** 1 Department of Animal Biotechnology, Dankook University, Cheonan 31116, Republic of Korea; sureshbiogenetic@gmail.com (S.S.K.); tpdus9860@naver.com (S.Y.J.); 2 Smart Animal Bio Institute, Dankook University, Cheonan 31116, Republic of Korea

**Keywords:** achyranthes japonica extract, cytokine production, growth performance, nutrient digestibility, milk thistle seed

## Abstract

The objective of this study was to assess the impacts of milk thistle seed (MTS) and Achyranthes japonica extract (AJE), both individually and in combination, on growth performance, nutrient digestibility, fecal score, fecal gas emissions, and cytokine responses in *n* = 120 crossbred [(Landrace Yorkshire) × Duroc] weaning piglets with an initial body weight of 6.53 ± 1.24 kg. Pigs were selected based on sex and randomly assigned to one of four dietary treatments for 6 weeks. The experimental diets were as follows: (1) CON—control/basal diet; (2) AJE (CON + 0.10% AJE); (3) MTS (CON + 0.10% MTS); and (4) CMB—combo feed (CON + 0.05% of AJE + 0.05% of MTS). Each treatment consisted of six replicates with five pigs (three ♀ and two barrows ♂) per pen. The incorporation of MTS has the potential to enhance (*p* < 0.05) the average daily gain in weaning pigs, particularly when used alone or in combination with AJE. But there were no significant effects or adverse effects observed on other growth parameters such as body weight, average daily feed intake, and gain-to-feed ratio. Also, there were no notable changes found in nutrient digestibility, fecal score, fecal gas emissions, or cytokine production. In summary, MTS and AJE, administered alone or in combination, reveal similar growth outcomes, suggesting that both additives could serve as potential options to improve animal performance without adverse effects.

## Simple Summary

Milk thistle seed (MTS) and Achyranthes japonica extract (AJE) both offer potential health benefits for pigs. MTS is rich in the bioactive compound and known for its hepatoprotective, anti-inflammatory, and antioxidant effects, promoting liver health and reducing inflammation. AJE, derived from the Achyranthes japonica (AJ) root, contains phytoecdysteroids and saponins, which are linked to anti-inflammatory, antioxidant, and growth-promoting properties. Earlier research suggests that MTS improves liver function and nutrient utilization, while AJE has shown benefits in enhancing growth performance and nutrient digestibility in pigs. To date, no study exists on examining the combined effects of MTS and AJE on weaning pigs. Thus, we aimed to assess the impacts of MTS and AJE, both individually and in combination, on growth performance, nutrient digestibility, fecal consistency, fecal gas emissions, and cytokine responses in pigs and found no significant effect observed for either additive.

## 1. Introduction

In intensive pig farming, particularly in high-density settings, completely protecting animals from exposure to various toxins, pro-inflammatory agents, and pharmaceuticals is virtually impossible. Current insights into farm animal nutrition highlight the importance of an optimal diet that not only meets essential nutrient requirements but also includes functional components that contribute biologically active substances to improve health and enhance nutrient utilization [1]. This holistic approach has gained prominence since the EU’s ban on antibiotic feed [2]. Among the various plant species suitable for livestock feed, milk thistle (*Silybum marianum* L.) is particularly noteworthy, especially its seeds known for hepatoprotective, anti-inflammatory, and antioxidant effects [3]. The predominant biologically active component of milk thistle seed (MSE) is silybin, constituting approximately 1.5–3.0% of its dry matter. Silymarin is a complex of seven flavonolignans, including silybin A, silybin B, isosilybin A, isosilybin B, silychristin A, silychristin B, and silydianin [4]. Of these, silybin is the most bioavailable material and silichristin the least [5]. These compounds have protective and regenerative effects on liver hepatocytes and modulate various cell signaling pathways, resulting in a decrease in pro-inflammatory mediators [6]. Also, it appeared to be more effective in improving weight gain, feed utilization, PUFA content in tissues, and water holding and antioxidant capacities in fattening pigs [7].

Achyranthes japonica, a member of the Achyranthes genus within the Amaranthaceous family, is primarily found in China, Korea, and Japan [8]. Its roots are known to contain several active compounds, including phytoecdysteroids, saponins, polysaccharides, inokosterone, and 20-hydroxyecdysone [9]. Research has shown that Achyranthes japonica extract (AJE) offers numerous health benefits, including anti-allergic, hepatoprotective, anti-inflammatory, alleviative, antioxidant, anti-cancer, and anti-arthritis effects [10]. For instance, Janocha et al. [11] demonstrated that incorporating AJE into the diet of broilers has positively affected their growth performance. Additionally, Liu et al. [12] found that AJE supplementation in the diets of growing pigs improves both growth performance and nutrient digestibility. Following this, Hossain et al. [13] found a significant impact on the growth performance (including average daily gain and average daily feed intake) and nutrient digestibility in growing pigs by the inclusion of 0.10% *Silybum marianum* seed extract. The above-mentioned studies with positive effects on using each additive individually led us to hypothesize that combining MTS and AJE additives could potentially yield even greater benefits in pigs and offer novel insights for the pig producers. To our knowledge, this is the first study to examine the combined effects of MTS and AJE on this age group pigs, and thus, we aimed to assess the impacts of MTS and AJE, both individually and in combination, on growth performance, nutrient digestibility, fecal consistency, fecal emissions, and inflammatory cytokine responses in weaning pigs.

## 2. Materials and Methods

The research protocol and procedures utilized in this study received ethical review and approval from the Institutional Animal Use and Care Committee (IAUAC) of Dankook University in South Korea (approval number DK-2-2323_4), in accordance with the ARRIVE guidelines.

### 2.1. Animals, Diet and Dietary Regimes

At the age of day 21, a total of 120 crossbred weaning piglets [(Landrace × Yorkshire) × Duroc] with an initial body weight of 6.53 ± 1.24 kg were selected and randomly assigned to one of four dietary treatment groups in a complete block cage for a period of 6 weeks. The experimental diets were as follows: (1) CO—control/basal diet; (2) AJE (CON + 0.10% AJE); (3) MTS (CON + 0.10% MTS); and (4) CMB—combo feed (CON + 0.05% of AJE + 0.05% of MTS). Each treatment consisted of six replicates with five pigs (three gilts ♀ and two barrows ♂) per pen. The mash form of the basal diet was formulated in three phases according to NRC [14] guidelines at the Swine Nutrition Feed Technology Lab (Table 1), and the test additives were sourced from Synergen Co. Ltd. (Bucheon, Republic of Korea). Before feeding, the basal diet and additives were thoroughly mixed using a DDK-801 feed mixer (Daedong Tech, Republic of Korea) and stored in pre-labeled bags. The temperature in the weaning barn was maintained at 24 °C. Self-feeders and water drinkers were fixed in the corner of each cage, allowing the pigs to have ad libitum access to both feed and water.

### 2.2. Sampling and Laboratory Analysis

Performance metrics including body weight (BW), average daily gain (ADG), average daily feed intake (ADFI), and gain-to-feed ratio (G: F) were measured at weeks 0–1, 2–3, 4–6, and the overall trial period. Seven days prior to fecal sample collection (i.e., from the end of week 5 to 6), all pigs were fed a diet containing 0.3% chromium oxide (Cr2O3), an indigestible marker to determine the nutrient digestibility of dry matter (DM), nitrogen (N), and energy (E). At the end of week 6, fresh fecal samples were collected from at least two pigs per pen (one male and one female) by rectal palpation, homogenized, and transported to the laboratory where they were stored at −20 °C until further analysis. Prior to the analysis, fecal samples were placed in a convection oven at 105 °C for three days. Afterwards, the samples were crushed and passed through a 1.2 mm sieve. The nutrient digestibility of DM (method 934.01) and N (method 984.13A-D) in both feed and fecal samples was analyzed according to AOAC [15]. Chromium was examined through UV absorption spectrophotometry (Shimadzu, UV-1201, Shimadzu, Kyoto, Japan). The E was determined by measuring the heat of combustion in the samples, using a bomb calorimeter (Parr 6100; Parr Instrument Co., Moline, IL, USA). N was determined by using a Kjeltec 2300TM Analyzer (Foss Tecator AB, Hoeganaes, Sweden). Apparent total tract digestibility (ATTD) was determined: ATTD = {1 [(Nf × Cd)/(Nd × Cf)], where Nf stands for nutrient concentration in feces, Nd stands for nutrient concentration in diet, Cr2O3d stands for chromium oxide concentration in diet, and Cf stands for chromium dioxide concentration in feces. Fecal consistency scores were recorded at the beginning and end of each experimental phase, following the assessment method described by Hu et al. [16]. At the end of week 6, two hundred grams of fresh fecal and urine samples were collected from two pigs per pen, homogenized, and placed in a container (3000 mL) with a small hole in the middle and sealed with plaster. Then the collected specimens were fermented at 25 °C for 24 h. Later, 100 mL of sample was taken from the headspace (approximately 2.0 cm) and shaken manually for 30s to assess the crust formation on the surface. Finally, the concentrations of ammonia (NH_3_), hydrogen sulfide (H_2_S), carbon dioxide (CO_2_), methyl mercaptans, and acetic acid were measured using a Multi-RAE Lite gas detection probe (model PGM-6208, RAE Systems, San Jose, CA, USA). At the end of week 10, two pigs per pen (one male and one female) were selected, and the blood sample was collected from their ear vein using K3EDTA evacuated tubes. Serum Alanine aminotransferase (ALT) and aspartate aminotransferase (AST) levels were measured using Product Nos. C009-2 and C0010-2 commercial kits, respectively. At once, peripheral blood samples were obtained from the experimental animals for cytokine analyses and the sera were frozen at −20 °C. The levels of TNF-α, IFN-γ, IL-1β, IL-6, IL-8, and IL-10 in the sera were determined using commercial ELISA kits specifically designed for porcine cytokine detection (R&D Systems Europe Limited, Abingdon, UK).

### 2.3. Statistical Analysis

Experimental data from the feeding trail were analyzed using the General Linear Model (GLM) procedure of the SAS software (9.0 version, SAS Inst. Inc., Cary, NC, USA). For all parameters (growth performance, nutrient digestibility, and fecal gas emission), pens served as the experimental unit. For fecal score and blood profile, individual pigs served as experimental units. Initial BW was used as a covariate for analyses of final BW. *p* < 0.05 was set as significant, while *p* < 0.10 was set as a trend.

## 3. Results

During the overall trial period, pigs fed MTS and CMB (Table 2) exhibited significantly increased (*p* < 0.05) ADG compared to those fed CON and AJE alone. However, there were no notable changes found on the rest of the performance measures including BW, ADFI, G: F. Also, the nutrient digestibility of DM, N, and E of pigs was not affected by dietary MTS and AJE, either individual or combined (Table 3). Furthermore, there were no notable changes found in their fecal consistency (Table 4), fecal gas emission (Table 5), and inflammatory cytokine (Table 6) production by the addition of MTS and AJE alone or in combination.

## 4. Discussion

Our results demonstrate that pigs fed MTS alone or in combination with AJE had higher ADG, which aligns with Liu et al. [12], who found similar results in finishing pigs by the addition of graded levels of AJE supplementation. The significant improvement in the ADG of pigs fed MTS alone or the combination with AJE is the most noteworthy finding of this study as these two additives were known for their active compound and possess antioxidant and anti-inflammatory properties and they might contribute to improved liver health and overall metabolic function in animals [17]. Also, the observed improvement in ADG, even in the absence of significant changes in other growth parameters such as BW, FI, or G: F, implies that MTS might have facilitated more efficient growth despite not increasing the overall feed intake. This aligns with earlier reports [18], which highlights the MTS’s ability to enhance nutrient absorption and metabolic activity in broilers without altering feed consumption [19,20,21]. However, the lack of a strong effect on BW, FI, and G:F ratios in the early weeks may indicate that the beneficial effects of MTS and AJE may take some time to manifest or that the doses used were insufficient to promote changes in these parameters.

The current research showed that the nutrient digestibility of DM, N, and E of pigs was not affected by dietary MTS and AJE, either individual or combined. This outcome was not in agreement with Nenova et al. [22], who found better weight gain in fattening pigs by the inclusion of MTS 10 g/day. However, some studies on MTS and other herbal additives showed improvements in nutrient digestibility due to their potential to modulate gut health, microbiota, and enzymatic activity [23]. For instance, Sun et al. [23] addressed that broiler fed diet supplemented with AJE has improved their apparent total tract digestibility of nitrogen and DM. A study by Oetting et al. [24] demonstrated that the use of phytogenic feed additives resulted in increased villus length [25] and decreased crypt depth, suggesting an enhancement in nutrient absorption. The proposed reason for the absence of significant effects on nutrient digestibility in this study implies several factors: (1) the pigs’ digestive systems may not have fully adapted to the dietary changes within trial period, or the inclusion rates of MTS and AJE (0.1%) may have been too low to produce significant effects. It is also possible that the composition of the basal diet or the physiological condition of the pigs, such as their gut health status during weaning, may have limited the ability of MTS and AJE to alter digestibility in a measurable way.

At the end of week 6, pigs fed MTS alone or in combination with AJE showed no significant differences in fecal gas emissions, suggesting that MTS and AJE did not influence gut microbiota or fermentation processes in the weanling pigs. Fecal scores, commonly used as indicators of intestinal health, reflect changes in gut function, with alterations in fecal consistency signaling either improvements or disruptions [26]. The lack of changes in fecal scores may indicate that the dietary additives did not alter microbial fermentation in the gastrointestinal tract during the trial period. Additionally, the absence of significant changes in fecal gas emissions, a key marker of fermentation activity, further supports this conclusion. To date, no studies have evaluated the effects of MTS and AJE blends in pigs; thus, direct comparisons could not be made. Thus, further research is needed to know the exact mechanism behind the lack of outcome. AST and ALT are key enzymes predominantly located in the liver [27], and their presence in the bloodstream is a well-established indicator of liver cell damage [28]. In the present study, pigs supplemented with MTS, either alone or in combination with AJE, exhibited no significant alterations in AST and ALT levels. This finding strongly suggests that these dietary additives neither induced hepatocellular damage nor exerted hepatoprotective effects over the 6-week feeding period. Moreover, cytokines play a pivotal role in regulating immune responses [29], particularly during the vulnerable post-weaning period when immune balance is critical for health and performance [30]. The lack of negative impact on both liver enzyme activity and cytokine production underscores the safety of MTS and AJE supplementation. Importantly, the data indicates that these additives did not provoke excessive immune activation or inflammatory responses.

## 5. Conclusions

The incorporation of MTS has the potential to enhance the growth performance of nursery pigs, particularly when used alone or in combination with AJE. Specifically, ADG was improved overall, but no significant effects and adverse effects were observed on other growth parameters such as BW, FI, and G: F, and there were no notable changes in digestibility, fecal parameters, gas emissions, or cytokine production. These findings suggest that the benefits of MTS and AJE on growth performance may be more evident after longer supplementation periods or under different conditions. Based on the findings, we infer that incorporating MTS alone or combined with AJE in nursery diet may serve as potential options to improve animal performance without adverse effects.

## Figures and Tables

**Table 1:** Composition of weaning pig diets.

Item	Basal Diet		
	Phase 1 (Week 0–1)	Phase 2 (Week 2–3)	Phase 3 (Week 4–6)
Ingredients (%)			
Extruded Corn	37.62	52.03	60.85
Soybean meal	18.25	16.68	19.05
Tallow	2.90	2.69	2.25
Fermented Soybean meal	5.00	4.00	3.00
DPP	5.00	3.00	2.00
Limestone	0.93	0.94	1.00
Salt	0.20	0.10	0.10
lactose	13.46	7.78	3.18
Sugar	3.00	3.00	3.00
whey protein	11.00	7.00	3.00
MDCP	1.10	1.30	1.35
Methionine (99%)	0.22	0.15	0.09
Lysine (78%)	0.51	0.65	0.57
Mineral mix ^1^	0.20	0.20	0.20
Vitamin mix ^2^	0.20	0.20	0.20
Choline (25%)	0.03	0.03	0.03
ZnO (80%)	0.38	0.25	0.13
Total	100.00	100.00	100.00
Calculated value			
Crude protein, %	20.00	18.00	18.00
ME (kcal/kg)	3450	3400	3350
Ca, %	0.80	0.80	0.80
P, %	0.60	0.60	0.60
Lys, %	1.60	1.50	1.40
Met, %	0.48	0.40	0.35
FAT, %	4.56	4.79	4.66
Lactose, %	20.00	12.00	5.00

^1^ Provided per kg diet: Fe, 100 mg as ferrous sulfate; Cu, 17 mg as copper sulfate; Mn, 17 mg as manganese oxide; Zn, 100 mg as zinc oxide; I, 0.5 mg as potassium iodide; and Se, 0.3 mg as sodium selenite. ^2^ Provided per kilograms of diet: vitamin A, 10,800 IU; vitamin D3, 4000 IU; vitamin E, 40 IU; vitamin K3, 4 mg; vitamin B1, 6 mg; vitamin B2, 12 mg; vitamin B6, 6 mg; vitamin B12, 0.05 mg; biotin, 0.2 mg; folic acid, 2 mg; niacin, 50 mg; D-calcium pantothenate, 25 mg.

**Table 2:** Effect of milk thistle seed and Achyranthes japonica extract on growth performance in weaning pigs ^1^.

Items	CON	AJE	MTS	CMB	SEM ^2^	*p* Value
Body weight, kg						
Initial	6.53	6.53	6.53	6.54	0.007	1.000
Week 1	8.53	8.55	8.59	8.59	0.02	0.999
Week 3	14.04	14.11	14.26	14.20	0.10	0.993
Week 6	25.42	25.73	26.27	26.01	0.13	0.752
Week 1						
ADG, g	286	287	294	292	3.10	0.264
ADFI, g	337	337	343	342	5.54	0.625
G: F	0.850	0.852	0.858	0.853	0.005	0.772
Week 3						
ADG, g	394	398	405	401	6.91	0.693
ADFI, g	540	543	546	543	9.91	0.981
G: F	0.729	0.732	0.742	0.738	0.006	0.564
Week 6						
ADG, g	542	553	572	563	5.86	0.093
ADFI, g	864	877	887	886	12.87	0.671
G: F	0.627	0.631	0.645	0.636	0.008	0.500
Overall						
ADG, g	450 ^b^	457 ^a,b^	470 ^a^	464 ^a^	3.24	0.024
ADFI, g	668	676	683	681	5.55	0.477
G: F	0.673	0.676	0.688	0.681	0.006	0.417

^1^ Abbreviations: (1) CON—control/basal diet; (2) AJE (CON + 0.10% AJE); (3) MTS (CON + 0.10% MTS); (4) CMB—combo feed (CON + 0.05% of AJE + 0.05% of MTS). ^2^ Standard error of means. ^a,b^ Means in the same row with different superscripts differ (*p* < 0.05).

**Table 3:** Effect of milk thistle seed and Achyranthes japonica extract on nutrient digestibility in weaning pigs ^1^.

Items, %	CON	AJE	MTS	CMB	SEM ^2^	*p* Value
Week 6						
Dry matter	85.01	87.86	88.93	87.66	0.83	0.421
Nitrogen	78.48	79.26	82.91	81.71	0.95	0.338
Energy	87.10	87.62	86.72	87.99	0.92	0.969

^1^ Abbreviations: (1) CON—control/basal diet; (2) AJE (CON + 0.10% AJE); (3) MTS (CON + 0.10% MTS); (4) CMB—combo feed (CON + 0.05% of AJE + 0.05% of MTS). ^2^ Standard error of means.

**Table 4:** Effect of milk thistle seed and Achyranthes japonica extract on gas emission in weaning pigs ^1^.

Items, ppm	CON	AJE	MTS	CMB	SEM ^2^	*p* Value
Week 6						
NH_3_	3.13	3.13	2.63	2.88	0.56	0.889
H_2_S	2.88	2.93	2.33	2.60	0.25	0.474
Methyl mercaptans	3.75	4.25	3.38	4.13	0.75	0.794
Acetic acid	8.38	7.50	7.38	9.13	1.29	0.717
CO_2_	10025	10175	9725	9975	537.61	0.931

^1^ Abbreviations: (1) CON—control/basal diet; (2) AJE (CON + 0.10% AJE); (3) MTS (CON + 0.10% MTS); (4) CMB—combo feed (CON + 0.05% of AJE + 0.05% of MTS). ^2^ Standard error of means.

**Table 5:** Effect of milk thistle seed and Achyranthes japonica extract on fecal score in weaning pigs ^1^.

Items	CON	AJE	MTS	CMB	SEM ^2^	*p* Value
Fecal score ^3^						
Initial	3.12	3.05	3.08	3.05	0.018	0.513
Week 1	3.04	3.04	3.00	3.01	0.019	0.846
Week 3	3.07	3.06	3.08	3.01	0.019	0.641
Week 6	3.05	3.01	3.01	3.04	0.020	0.916

^1^ Abbreviations: (1) CON—control/basal diet; (2) AJE (CON + 0.10% AJE); (3) MTS (CON + 0.10% MTS); (4) CMB—combo feed (CON + 0.05% of AJE + 0.05% of MTS). ^2^ Standard error of means. ^3^ Fecal score = 1—hard, dry pellet; 2—firm, formed stool; 3—soft, moist stool that retains shape; 4—soft, unformed stool that assumes shape of container; 5—watery liquid that can be poured.

**Table 6:** Effect of milk thistle seed and Achyranthes japonica extract on blood profile and pro-inflammatory cytokine level in weaning pigs ^1^.

Items, U/L	CON	AJE	MTS	CMB	SEM ^2^	*p* Value
Week 6						
AST	73.33	69.83	61.67	64.17	3.24	0.601
ALT	78.83	74.33	67.67	69.83	1.80	0.120
TNF-α	173.19	157.10	141.06	142.53	5.72	0.158
IFN-γ	4.51	3.67	2.82	3.45	0.27	0.190
IL-1β	71.68	68.90	63.49	58.86	4.13	0.725
IL-6	290.96	271.74	250.83	260.40	11.04	0.636
IL-8	64.63	59.82	51.16	55.12	3.09	0.471
IL-10	7.20	5.82	4.36	5.97	0.46	0.193

^1^ Abbreviation: (1) CON—control/basal diet; (2) AJE (CON + 0.10% AJE); (3) MTS (CON + 0.10% MTS); (4) CMB—combo feed (CON + 0.05% of AJE + 0.05% of MTS). ^2^ Standard error of means.

## Data Availability

The data presented in this study are available on request from the corresponding author.
